# Attempted Depletion of Passenger Leukocytes by Irradiation in Pigs

**DOI:** 10.1155/2011/928759

**Published:** 2011-12-18

**Authors:** Hao-Chih Tai, Xiaocheng Zhu, Yih Jyh Lin, Hidetaka Hara, Mohamed Ezzelarab, Michael Epperley, Mubina A. Quader, David K. C. Cooper

**Affiliations:** ^1^Thomas E. Starzl Transplantation Institute, University of Pittsburgh Medical Center, Pittsburgh, PA 15261, USA; ^2^Department of Surgery, National Taiwan University Hospital and College of Medicine, Taipei 100, Taiwan; ^3^Department of Radiation Oncology, University of Pittsburgh Medical Center, Pittsburgh, PA 15261, USA

## Abstract

Allograft/xenograft rejection is associated with “passenger leukocyte” migration from the organ into recipient lymph nodes. In Study 1, we attempted to deplete leukocytes from potential kidney “donor” pigs, using two regimens of total body irradiation. A dose of 700 cGy was administered, followed by either 800 cGy (“low-dose”) or 1,300 cGy (“high dose”) with the kidneys shielded. Neither regimen was entirely successful in depleting all leukocytes, although remaining T and 8 cell numbers were negligible. Study 2 was aimed at providing an indication of whether near-complete depletion of leukocytes had any major impact on kidney allograft survival. In non-immunosuppressed recipient pigs, survival of a kidney from a donor that received high-dose irradiation was compared with that of a kidney taken from a non-irradiated donor. Kidney graft survival was 9 and 7 days, respectively, suggesting that depletion had little impact on graft survival. The lack of effect may have been related to (i) inadequate depletion of passenger leukocytes, thus not preventing a direct T cell response, (ii) the presence of dead or dying leukocytes (antigens), thus not preventing an indirect T cell response, or (iii) constitutive expression of MHC class II and B7 molecules on the porcine vascular endothelium, activating recipient T cells.

## 1. Introduction

Passenger leukocytes (contained within a transplanted organ and which migrate from the graft into the lymphoid tissues of the recipient) have complex dual roles in determining the outcome after transplantation (Tx). Donor-derived passenger leukocytes can initiate graft rejection in some settings, whereas in others they contribute to graft acceptance [1–3]. In a small animal model, *donor* pretreatment with sublethal total body irradiation (TBI) or antilymphocyte globulin resulted in significant prolongation of heart allograft survival [4]. In animal models of intestinal Tx, passenger leukocytes in the intestine have a lineage profile that predisposes to graft-versus-host disease, which can be eliminated by *ex vivo* irradiation (10 Gy) of mature lymphoid elements from the bowel allografts [5]. In contrast, lethal TBI of the donor resulted in loss of ability to achieve tolerance in a pig kidney alloTx model [6]. 

The absolute number of MHC class II^+^ dendritic cells present in a donor organ is a poor predictor of graft outcome, and survival of solid organ allografts is more closely related to the density of the donor dendritic cell network within the graft [7]. Prior irradiation of the potential organ donor leads to killing of the passenger leukocytes. Complete depletion of passenger leukocytes would likely prevent the direct T-cell response, though not the indirect, but might impact subsequent kidney graft survival.

The primary aim of the present study, therefore, was to determine whether a sufficient dose of irradiation could be administered to the “donor” pig to kill the leukocytes without seriously damaging the kidney (to be transplanted). The secondary aim was to determine whether complete or near-complete depletion of passenger leukocytes delayed rejection after kidney Tx.

Total body irradiation of the donor of 700 cGy with a further 800 cGy or 1,300 cGy with the kidneys shielded proved insufficient to deplete all leukocytes. However, the function of the irradiated kidney and any possible protection from rejection was tested by Tx into a recipient pig without immunosuppressive therapy.

## 2. Methods

### 2.1. Animals

Outbred large white landrace pigs (Wally Whippo, Enon Valley, PA), aged 4–10 months, weighing 20–50 kg, were used to assess two irradiation regimens (*n* = 3 in each group) and as sources of kidneys (*n* = 3) for Tx into recipients (*n* = 3). All animal care and procedures were in accordance with the *Principles of Laboratory Animal Care* formulated by the National Society for Medical Research and the *Guide for the Care and Use of Laboratory Animals* prepared by the Institute of Laboratory Animal Resources and published by the National Institutes of Health (NIH publication number 86-23, revised 1985).

### 2.2. Irradiation

Pigs were sedated with ketamine (7.5 mg/kg IV) and received TBI of 700 cGy (350 cGy to each side) and then, on the same occasion, received a further 800 or 1300 cGy (400 or 650 cGy to each side) with both kidneys shielded. The dosages were determined from a review of the literature [6, 8–10]. The pigs were irradiated using a 6 MV photon beam from a Varian CLINAC 600C linear accelerator (Varian Medical System, Palo Alto, CA). TBI was performed by placing the pigs on the floor at an extended distance of 230 cm from the source and using a collimated beam of 40 × 40 cm^2^, with the beam pointing downward towards the floor. This approach accommodated the entire body of the pig in the irradiation area. The pigs were radiated sequentially in right and left lateral decubitus positions with the longitudinal axis of the pigs placed along the horizontal axis of the radiation field. The prescribed dose was delivered to the midbody using equally weighted parallel opposed lateral fields. A dose rate of 250 MU/min was used during irradiation. Dose rates at different depths were measured under irradiation conditions to calculate radiation time and monitor units. The thickness of the pigs at midbody varied from 15–21 cm. To accomplish shielding of the kidneys, the positions of the kidneys were identified using ultrasound imaging and their sites indicated by marking the skin. Both kidneys were protected from the second dose of irradiation by lead shields.

### 2.3. Surgical Procedures

#### 2.3.1. Insertion of Intravenous Catheters

In all pigs, the right internal and external jugular veins were cannulated with silastic catheters, and the cannulae tunneled under the skin to the back, where they were brought out through the skin. These allowed access for drug administration and blood withdrawal for monitoring of renal function, tacrolimus levels (where indicated), and *in vitro* assays throughout the course of the experiment.

#### 2.3.2. Kidney Transplantation

Life-supporting kidney Tx was carried out to leave no doubt that the survival of the pig depended on the function of the transplanted kidney. The right kidney of the donor pig was perfused with 2L cold (4°C) University of Wisconsin solution before nephrectomy. The recipient pig underwent right nephrectomy and kidney Tx. End-to-side anastomoses were carried out between the donor renal artery and recipient aorta and the donor renal vein and recipient inferior vena cava. The donor ureter was implanted into the recipient bladder. A double-J stent was placed from the pelvis of the kidney graft to the recipient bladder to maintain patency of the ureteric anastomosis. When the kidney graft had shown good urine output for >30 min, the native contralateral ureter was doubly ligated. Graft function was monitored daily by serum creatinine. A serum creatinine of >884 *μ*mol/L (>10 mg/dL) indicated failure of the kidney.

#### 2.3.3. Immunosuppressive Therapy

In one recipient pig, during the operative procedure (Day 0) immunosuppression was initiated with tacrolimus (Prograf, Astellas Pharma US, Deerfield, IL) at 0.1 mg/kg/day by i.v. infusion. Thereafter, tacrolimus was given i.m. at a dose of approximately 0.05 mg/kg ×2 daily, the dose being adjusted to maintain a 12 h blood trough level of 10–15 ng/mL. Blood levels of tacrolimus were monitored daily to ensure the dosage was optimum. This therapy was continued for 42 days. No other immunosuppressive therapy was administered.

### 2.4. Measurement of White Blood Cell (WBC) Counts

Complete WBC counts on pig blood were carried out using automated methodology at the University of Pittsburgh Medical Center Central Laboratory, Presbyterian Hospital, Pittsburgh, PA.

### 2.5. Histopathologic Examination

After euthanasia of the pigs, necropsies were performed through a midline abdominal incision. Kidney specimens were taken at necropsy, placed in formalin, stained with hematoxylin and eosin, and examined microscopically. Kidney allograft rejection (acute cellular rejection) was scored by standard pathologic criteria according to the cooperative clinical trials in transplantation (CCTT) classification system and incorporated into the revised Banff criteria [11, 12].

### 2.6. Isolation and Storage of Peripheral Blood Mononuclear Cells (PBMC)

PBMC from donor, recipient, and third-party pigs were isolated from heparinized blood by centrifugation over Ficoll-Paque PLUS (Amersham Biosciences, Piscataway, NJ), as described previously [13]. The total mononuclear cell fraction was washed twice with PBS (Gibco, Grand Island, NY) and then resuspended in FACS buffer (PBS containing 1% BSA and 0.1% NaN_3_) for flow cytometry or in mixed lymphocyte reaction (MLR) medium (including 10% controlled processed serum replacement type 3 [CPSR-3], Sigma, St Louis, MO) for MLR assay.

Donor and third-party pig PBMCs were stored in 10% DMSO RPMI with 20% FBS and kept at −80°C for 24 h before being stored in liquid nitrogen. For MLR, the cryopreserved donor and third-party pig PBMCs were thawed by a rapid rewarming procedure in a water bath at 37°C and washed with 10% FBS RPMI medium.

### 2.7. Flow Cytometry

Fresh heparinized pig blood (400 *μ*L) was lysed ×2 using ACK lysing solution to obtain pig WBCs, which were washed with PBS, resuspended with FACS buffer, and aliquoted into FACS tubes (80 *μ*L/tube). Detection of T, B, CD4^+^, and CD8^+^ cells was determined by incubating the pig WBC with, respectively, 10 *μ*L mouse anti-pig CD3*ε*-FITC mAb at a dilution of 1 : 64, mouse anti-human CD21-PE mAb at 1 : 16, mouse anti-pig CD4a-PE mAb at 1 : 16, or mouse anti-pig CD8a-PE mAb at 1 : 64. (All mAbs were obtained from BD Biosciences Pharmingen, Franklin Lakes, NJ). The samples were washed twice, and the cells resuspended with 200 *μ*L of FACS buffer. Data acquisition was performed with FACScan (Becton Dickinson, Mountain View, CA) using Cell Quest software. Dead cells were excluded from the analysis by side-scatter and propidium iodide staining (10 *μ*L added to each sample prior to FACS analysis). Data analysis was carried out using WinMDI software.

### 2.8. Mixed Lymphocyte Reaction (MLR)

PBMC were isolated from fresh heparinized pig blood, as described above, to a concentration of 4 × 10^6^ cells/mL. Pig PBMCs (0.4 × 10^6^ cells/well) were incubated with an equal number of irradiated stimulator cells (0.4 × 10^6^ cells/well) in 10% CPSR-3 RPMI medium at 37°C and 5% CO_2_ using 96-well round-bottom plates (Corning, New York, NY) [13]. Stimulator cells were irradiated with 2,500 cGy in a cesium^137^ irradiator. Each assay was performed in triplicate. After 4 days, 10 *μ*L of 3H-thymidine-labeling medium (1 *μ*Ci/well; New England Nuclear, Boston, MA) was added to each well. Cells were harvested after 18 h on glass-fiber filter mats with a cell harvester and were analyzed by beta counting on a liquid scintillation counter. Responder pig PBMCs were stimulated with autologous, donor, and third-party pig PBMC. The results were recorded as stimulation index (ratio of the response compared to that of autologous cells).

## 3. Experimental Groups

### 3.1. Study 1: Experiments to Determine Optimum Dose of Irradiation to Deplete Leukocytes without Damaging the Kidneys

An attempt was made to determine (i) the optimum dose of irradiation that would deplete leukocytes without damaging the kidneys, and (ii) the optimum period that should elapse before the kidneys should be excised for Tx. Pigs received either low-dose (700 + 800 cGy; *n* = 3) or high-dose (700 + 1300 cGy, *n* = 3) irradiation. All pigs were euthanized 60 or 72 hours after irradiation for examination of the kidneys.

### 3.2. Study 2: Experiments to Determine the Effect of Donor Pre-Transplant Total Body Irradiation on Kidney Allograft Survival

Life-supporting kidney Tx was carried out in the following experiments.

**Experiment 1**

A kidney from a *nonirradiated* donor into a recipient immunosuppressed with tacrolimus for 42 days.

**Experiment 2**

A kidney from a *nonirradiated* donor into a nonimmunosuppressed recipient.

**Experiment 3**

A kidney from an *irradiated *donor (700 + 1300 cGy carried out 72 h previously) into a nonimmunosuppressed recipient.

In Experiments 2 and 3, the donor and recipient pairs were reported *not* to be siblings.

## 4. Results

### 4.1. Study 1: Experiments to Determine Optimum Dose of Total Body Irradiation to Deplete Leukocytes without Damaging the Kidneys

**Figure 1:**
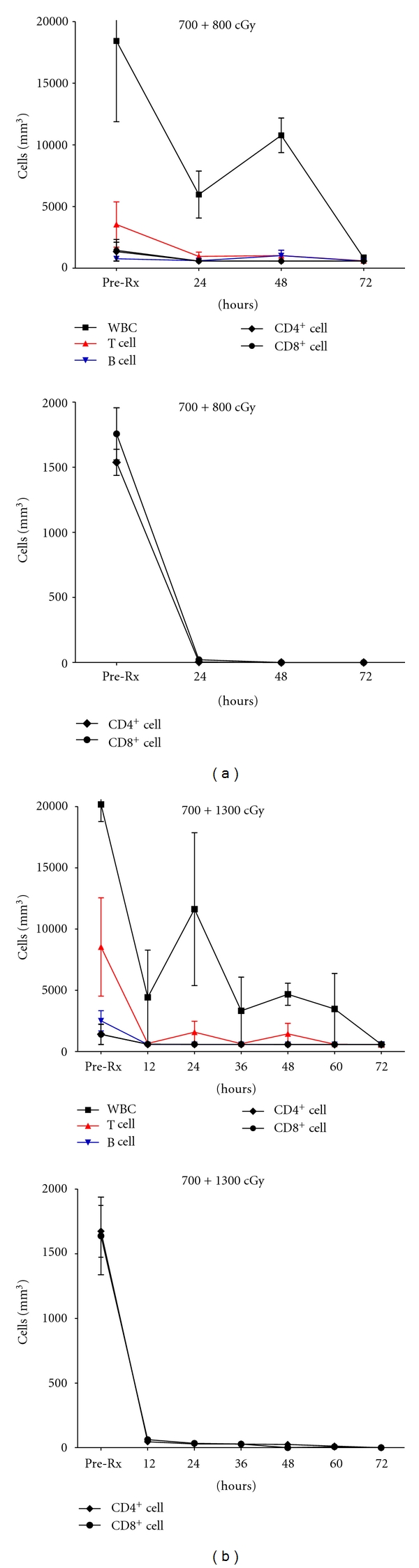
Cell counts in the blood of pigs receiving (a) low-dose or (b) high-dose irradiation. (a) Mean total WBC, T and B cell counts (*left upper*), and CD4^+^ and CD8^+^ cell counts (*left lower*) of 3 pigs receiving WBI of 700 cGy and a further 800 cGy with both kidneys shielded. (b) Mean total WBC, T and B cell counts (*right upper*), and CD4^+^ and CD8^+^ cell counts (*right lower*) of 3 pigs receiving WBI of 700 cGy and a further 1300 cGy with both kidneys shielded. Although T and B cell counts decreased rapidly to <100 cells/mm^3^ within 24 h after irradiation, the total WBC count did not reach such a low number for 72 h. The remaining WBCs were largely polymorphonuclear neutrophils.

During the 60–72 h after irradiation and before euthanasia, no pig experienced any vomiting or other obvious side effects from the irradiation, and no hematuria was observed. The cell counts in the blood of all pigs receiving either low-dose (*n* = 3) or high-dose (*n* = 3) irradiation indicated that the number of WBC, T cells, and B cells decreased significantly within 24 h of irradiation (Figure 1). The CD3^+^, CD4^+^, CD8^+^, and B cell counts in the blood were all <100/mm^3^ 24 h after irradiation. By 72 h, there were almost no cells in the blood, and the occasional cells that remained were polymorphonuclear neutrophils.

**Figure 2:**
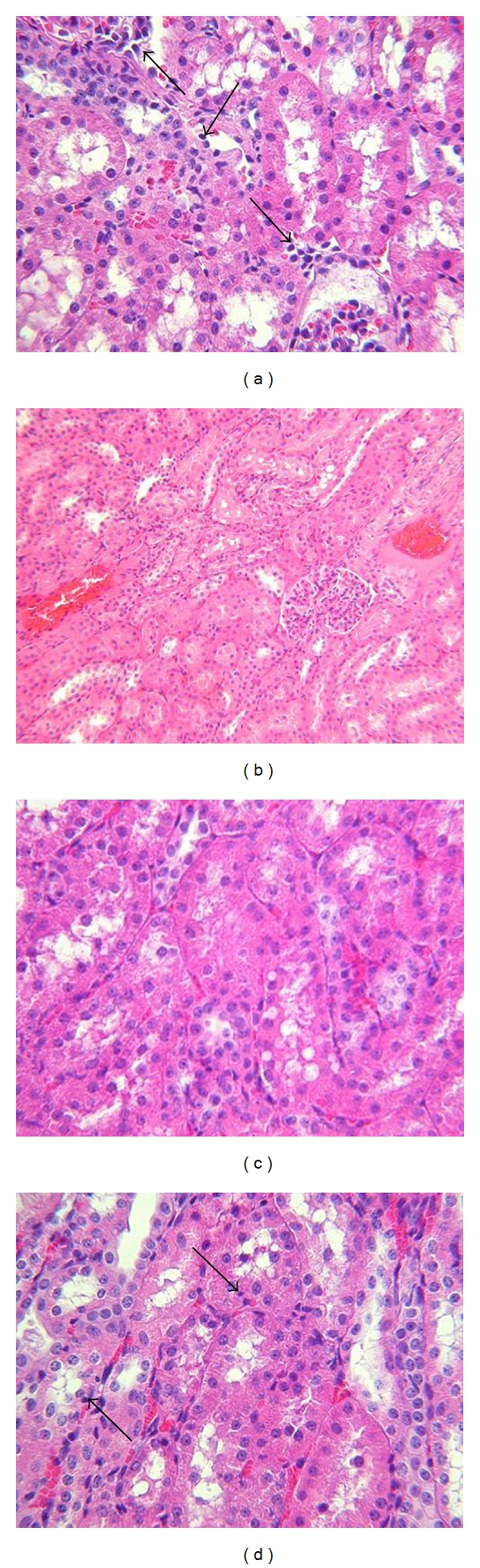
Histopathology of kidneys from a pig that received WBI 72 h previously of 700 cGy and a further 800 cGy with both kidneys shielded (H&E, magnification ×100). (a) A few passenger leukocytes can be detected in the interstitium (arrows). (b) Congestion is present in some peritubular blood vessels. (c) Tubular vacuolization and cloudy swelling of tubular cells are present. (d) Tubular epithelial apoptosis can be detected (arrows).

**Figure 3:**
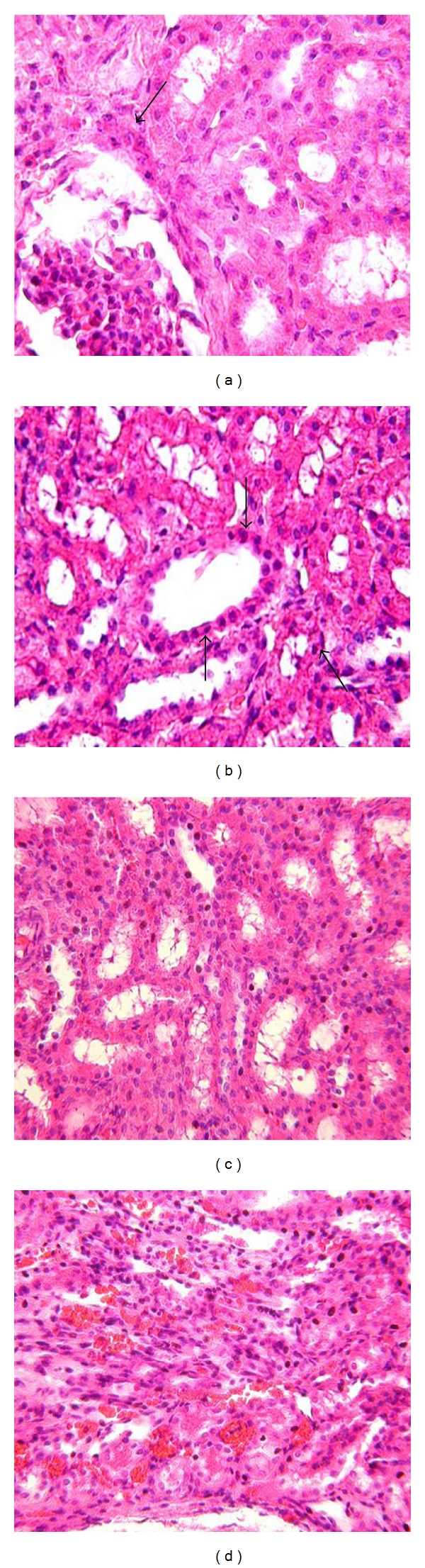
Histopathology of a kidney from a pig that received WBI 72 h previously of 700 cG and a further 1300 cGy with both kidneys shielded (H&E, magnification ×100). (a) A few passenger leukocytes can be detected in the interstitium (arrow). (b) A few apoptotic cells can be detected (arrows). (c) Minor hemorrhages are seen in the interstitium of medulla. (d) Congestion of peritubular blood vessels and interstitial edema can be seen.

The histopathologic examination of the kidneys of pigs that had received low-dose irradiation showed occasional residual passenger leukocytes, with congestion of blood vessels, tubular vacuolization, and some tubular epithelial apoptosis (Figure 2). The kidneys of pigs that had received high-dose irradiation showed very rare leukocytes, apoptotic cells, and minor hemorrhages in the medulla (Figure 3).

We concluded that low-dose irradiation depleted most, but not all, leukocytes in the kidneys. High-dose irradiation caused virtually complete depletion of leukocytes within 72 h and more apoptosis of cells, but also slightly more congestion and hemorrhage in the kidneys (Figure 3(d)), suggesting some radiation injury, possibly related to inadequate protection of the kidneys during the procedure. The period that had elapsed between irradiation and microscopic examination of the kidneys (60 h or 72 h) did not appear to influence the histopathologic features.

As there was a minimal number of leukocytes remaining in the blood of the donor 72 h after high-dose irradiation, and as we hypothesized that any remaining cells might be dying, we determined to test whether the donor treatment had had any effect on kidney survival after Tx. For this study, the higher dose of irradiation was selected, that is, 700 cGy and a further 1300 cGy with both kidneys shielded, which was carried out very carefully to avoid injury to the kidneys. Seventy-two hours was selected as the period that would elapse before kidney excision and Tx. 

### 4.2. Study 2: Experiments to Determine the Effect of Donor Pre-Transplant Total Body Irradiation on Kidney Allograft Survival

#### 4.2.1. Life-Supporting Kidney Tx from a Nonirradiated Donor into an Immunosuppressed Recipient

**Figure 4:**
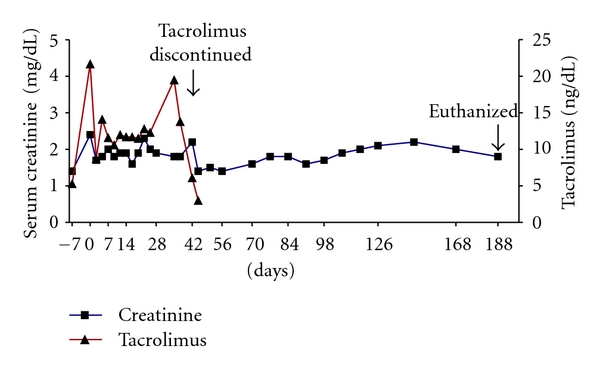
Life-supporting kidney Tx was carried out from a nonirradiated donor into a recipient immunosuppressed for 42 days with tacrolimus. Efforts were made to maintain the 12 h blood trough level between 10–15 ng/mL. Serum creatinine levels remained between 132.6–176.8 *μ*mol/L (1.5–2.0 mg/dL) for almost 5 months after discontinuing tacrolimus. The pig was euthanized electively 188 days after kidney Tx.

This experiment was carried out to document that the surgical technique was successful. To allow differentiation between graft failure from surgical complication and rejection, tacrolimus was administered for 42 days to prevent rejection in the early post-Tx course. The kidney clearly remained viable throughout the period of followup since the serum creatinine was maintained <176.8 *μ*mol/L (<2 mg/dL) for almost 5 months after discontinuation of tacrolimus immunosuppression (Figure 4). The pig was euthanized electively 188 days after kidney Tx. At all time intervals (both before and after Tx), the MLR showed a very low stimulation index to donor-specific stimulators (not shown). At necropsy, histological examination of the transplanted kidney showed minimal abnormality.

#### 4.2.2. Life-Supporting Kidney Tx from a Nonirradiated Donor into a Nonimmunosuppressed Recipient

**Figure 5:**
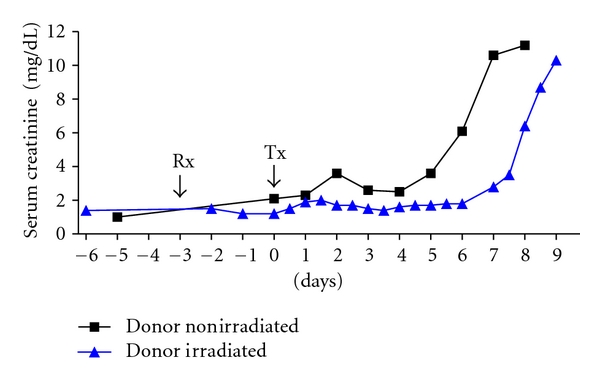
Life-supporting kidney Tx was carried out from (i) a nonirradiated donor (squares) and (ii) an irradiated donor (triangles) into nonsibling recipients without immunosuppressive therapy. The serum creatinine levels of the pigs rose to >884 *μ*mol/L (>10 mg/dL) within 7 and 9 days, respectively. Rx: day of irradiation to donor (day −3); Tx: day of kidney Tx (day 0).

The serum creatinine increased to >884 *μ*mol/L (>10 mg/dL) within 7 days (Figure 5). MLR showed a moderately high response before Tx (stimulation index 18) with a reduced response at the time of rejection (stimulation index 7) (not shown). Histopathology showed the typical features of acute cellular rejection (not shown).

#### 4.2.3. Life-Supporting Kidney Tx from an Irradiated Donor into a Nonimmunosuppressed Recipient

Following irradiation, the data were consistent with those observed in the preliminary irradiation study (Figure 1). By the day of kidney Tx (day 0–72 h after TBI), there were virtually no T, B, CD4^+^, or CD8^+^ cells remaining in the donor blood. After Tx, however, the serum creatinine increased to >884 *μ*mol/L (>10 mg/dL) within 9 days (Figure 5). Both before irradiation and at the time of graft failure, the MLR showed a very low response to donor-specific PBMC (not shown, suggesting significant inbreeding in some members of the herd). Histopathology of the graft showed the typical features of acute cellular rejection (not shown). No hematuria was observed in the donor pig after irradiation or in the recipient after kidney Tx.

## 5. Discussion

To determine the dose of irradiation required to deplete all passenger leukocytes, rodent models will not provide the data required since the dose of irradiation likely to achieve the desired effect will be different from that in large animals. It is known that unfractionated doses of irradiation have a greater effect than fractionated doses in destroying all of the passenger leukocytes [14, 15]. In humans, a “standard” 10 Gy single dose of TBI appears to be consistently more toxic than fractionated TBI regimens delivering from 12 to 15 Gy [14, 15]. It is also known that animals will survive for approximately 14 days before succumbing from the effects of a lethal dose of irradiation [16]. Mezrich et al. [6]. found that some pigs did not tolerate 1,500 cGy of TBI and developed renal failure secondary to irradiation toxicity; a dose of 1,000 cGy was well tolerated.

The irradiated pigs in the present study were euthanized 60–72 h after irradiation to prevent irradiation-related complications. All cell types measured in the blood (WBC, T, CD4^+^, CD8^+^, B) were significantly depleted by both dosages of irradiation tested and particularly by the higher dose (700 + 1300 cGy). By 72 h after TBI, T and B lymphocyte depletion from the blood was total except for an occasional polymorphonuclear leukocyte. The dose of irradiation selected may not have depleted all T and B cells in the thymus and/or spleen and/or bone marrow; one of the limitations of our study is that we did not monitor for leukocytes in these organs. At the higher dose of irradiation, minor injury to the kidneys was seen on histological examination, possibly related to inadequate protection of the kidneys. The pathophysiology of radiation injury of the kidney is poorly understood, and its presentation can be acute and irreversible or subtle, with a gradual progressive dysfunction over years [17].

We would conclude that perhaps neither of the irradiation regimens used was totally successful in depleting all passenger leukocytes in the donor kidney. Higher doses of irradiation, however, might have been associated with increased morbidity in the donor pig, as reported by Mezrich et al. [6] (and were, in fact, opposed on humane grounds by the Institutional Animal Care and Use Committee). We were, therefore, not able to test any higher doses of irradiation in this study. Nevertheless, despite the possibility that depletion might have been incomplete, we decided to test the effect of the donor irradiation by kidney Tx.

A control kidney Tx in a recipient that received a 42-day course of tacrolimus in the absence of irradiation to the donor indicated that the kidney transplant procedure was technically successful and that, when a kidney was transplanted from an outbred pig from the same herd, operational tolerance developed. This result suggested that either (i) the donor and recipient pigs were closely related, for example, siblings, or (ii) the herd was sufficiently inbred to allow kidney Tx in the absence of graft rejection, particularly in the presence of a 42-day course of tacrolimus.

The pig is known to be a relatively easy Tx model in which to develop tolerance to a *kidney *allograft if a short course of immunosuppressive therapy is administered. Kidney allografts between the Massachusetts General Hospital (NIH) [18] MHC two-haplotype *class I mismatched* miniature swine that receive a 12-day course of cyclosporine are uniformly accepted [19]. Using the same regimen, a renal allograft across a MHC two-haplotype *full-mismatch* barrier is rejected, but tolerance can be achieved if high-dose tacrolimus replaces cyclosporine [20]. In the present study (in which the MHC profiles of the donor-recipient pairs were not investigated) the long-term graft survival in Experiment 1 indicates that operational tolerance was achieved by a 42-day course of tacrolimus at standard dosage.

When no immunosuppressive therapy was administered to pigs that received a kidney from either a nonirradiated donor or irradiated donor, acute cellular rejection developed within 7–9 days. In both cases, the donor and recipient pigs were *not* siblings (but the MHC profile was not known, and the MLR was weak between one donor-recipient pair). This indicated that irradiation of the donor (in the dosage administered) did not prevent rejection (possibly because depletion of donor passenger leukocytes had been incomplete or because antigens from dead leukocytes were presented by recipient antigen-presenting cells). Furthermore, in the absence of a course of tacrolimus, operational tolerance was not achieved after kidney Tx between members of this herd.

The present study also suggests that MLR data in pigs may not be indicative of graft outcome, as illustrated by Experiment 2 (in which the pre-Tx response on MLR was high) and Experiment 3 (in which it was very low), and yet graft failure from acute cellular rejection developed in similar time frames (although TBI to the donor may have influenced the outcome). 

In view of the difficulty in completely depleting passenger leukocytes in the donor and the lack of any obvious difference in survival between the two kidney allografts, we did not feel that there was any purpose in pursuing the project further.

It would be possible to excise the kidney from the donor pig and irradiate it *ex vivo. *However, there might be several problems with this approach. Lymphocyte numbers significantly decrease after irradiation of pigs *in vivo* or of pig blood *in vitro* in a dose-dependent manner, but reduction of lymphocyte numbers after irradiation *in vivo* proceeds much faster than after irradiation *in vitro* [21]. Furthermore, a significantly different response to irradiation is achieved *in vivo* than* in vitro *or *ex vivo*. To have an optimum effect, irradiation depends on high oxygen content in the tissues [22, 23]. There is likely to be a low oxygen partial pressure in a cold *ex vivo* kidney, and this would reduce the effect of irradiation on the passenger leukocytes within the kidney. In view of a lack of background and experience in this approach, it would probably require several experiments to elucidate the optimum dosage if the organ was to be subjected to irradiation *ex vivo*.

Combining irradiation of the donor with the administration of a cytotoxic agent, for example, cyclophosphamide (although our evidence is that this drug is ineffective in pigs [24]) might result in more complete depletion of leukocytes. In the present study, monitoring leukocyte depletion in the thymus, spleen, or bone marrow would have provided a more accurate picture of the number and phenotype of the leukocytes remaining viable.

In summary, TBI at the doses administered was well tolerated by the pigs, but was not completely successful in depleting all passenger leukocytes, and this may not have completely prevented a direct T-cell response. Furthermore, we could not exclude presentation of donor antigens from dead or dying passenger leukocytes transplanted with the graft by recipient antigen-presenting cells, and therefore the indirect response may have been active.

Although kidney Tx was carried out in only one pig that had received a kidney from an irradiated donor, graft survival was only minimally prolonged compared with that of a nonirradiated kidney. We conclude that the irradiation regimens used in the present study were insufficient to deplete all leukocytes and did not prolong kidney graft survival after Tx. Further investigation of means of effectively depleting passenger leukocytes in large animals is required. The study did not rule out that TBI of the donor may be *detrimental* to kidney graft survival, though the results do not support this conclusion.

However, one other factor has to be considered in assessing the results. Porcine endothelial cells express MHC class II molecules *constitutively*, at least in some vascular beds [25, 26], indicating that they will be recognized by CD4^+^ T cells. They also constitutively express porcine B7 molecules [27], indicating that they may activate CD4^+^ cells just as efficiently as hematopoietic cells. It may be, therefore, that, even in the complete absence of passenger leukocytes in a transplanted pig organ allograft, acute rejection may still develop unless immunosuppressive therapy is administered for a period of time.

If pigs are to be used for these studies, it would be essential to have donors and recipients known to be of different MHC, and some immunosuppressive therapy would appear to be required, though the intensity and duration of the therapy would require careful investigation, making the model a difficult one. As it is so relatively easy to induce tolerance in a pig (in contrast, e.g. to a nonhuman primate or human) it may be that the pig is not the ideal animal model in which to explore this phenomenon.

## Abbreviations

MLR: Mixed lymphocyte reaction
PBMCs: Peripheral blood mononuclear cells
TBI: Total body irradiation
Tx: Transplantation
WBC: White blood cells.
